# Stress transfer outpaces injection-induced aseismic slip and triggers seismicity

**DOI:** 10.1038/s41598-023-43760-0

**Published:** 2023-10-03

**Authors:** Yuyun Yang, Hongfeng Yang, Jinping Zi

**Affiliations:** 1 Earth and Environmental Sciences Programme, The Chinese University of Hong Kong, Shatin, Hong Kong; 2 Shenzhen Research Institute, The Chinese University of Hong Kong, Shenzhen, China; 3 Institute of Environment, Energy and Sustainability, The Chinese University of Hong Kong, Shatin, Hong Kong

**Keywords:** Seismology, Geophysics

## Abstract

As concerns rise over damaging earthquakes related to industrial activities such as hydraulic fracturing, geothermal energy extraction and wastewater disposal, it is essential to understand how subsurface fluid injection triggers seismicity even in distant regions where pore pressure diffusion cannot reach. Previous studies suggested long-range poroelastic stressing and aseismic slip as potential triggering mechanisms. In this study, we show that significant stress transfer far ahead of injection-induced aseismic slip can travel at much higher speeds and is a viable mechanism for distant earthquake triggering. It could also explain seismicity migration that is much faster than aseismic slip front propagation. We demonstrate the application of these concepts with seismicity triggered by hydraulic fracturing operations in Weiyuan shale gas field, China. The speed of stress transfer is dependent on the background stress level and injection rate, and can be almost an order of magnitude higher than that of the aseismic slip front.

## Introduction

Fluid injection can trigger seismicity through various mechanisms including pore pressure diffusion^1,2^, aseismic slip^3^ and poroelastic stressing^4,5^. The speeds and spatial extents at which these processes occur also vary significantly: diffusion is dependent on the hydraulic diffusivity; aseismic slip can outpace diffusion after an initial period of acceleration^6^, and poroelasticity dominates at large distances from the injection site^7^. Often, the migration of seismicity is attributed to the advancement of the aseismic slip front^8–11^.

**Figure 1:**
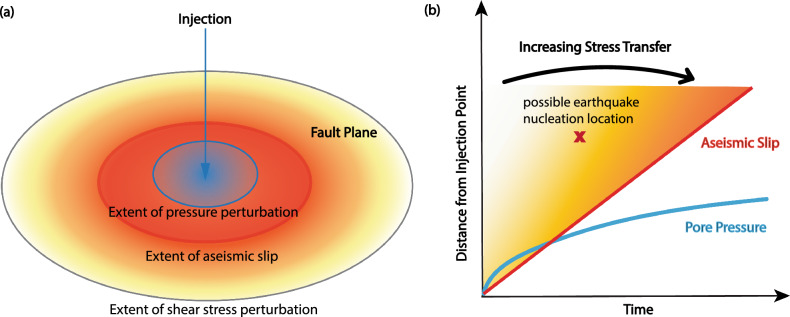
(**a**) Schematic diagram showing the extent of influence of pore pressure, aseismic slip and shear stress perturbations on a fault at a moment sometime after injection begins. (**b**) Migration speed of pore pressure (blue) and aseismic slip (red). A range of Coulomb failure stress transfer of varying magnitudes is possible outside of the aseismic slip front, which can be considered as the front of maximum stress perturbation. Stress transfer increases as we get closer to the slip front, but even far ahead of the slip front, small magnitudes of stress transfer are already being radiated and could have an impact on distant triggering of seismicity. We have marked a location where an earthquake could potentially nucleate, due to such radiated stress transfer, even though the location falls outside of the slip front.

However, elastic shear stress transfer radiating from and far ahead of injection-induced aseismic slip can also be very significant at large distances from the injector and has not been well studied. We can consider the advancement of the aseismic slip front as the propagation of the maximum shear stress perturbation. However, stress perturbations of much smaller magnitudes are already propagating at large distances ahead of the slip front itself. If certain highly stressed frictional asperities are already present, they could rupture given just a small stress increase before the aseismic slip front actually arrives. Figure 1a shows a schematic diagram of the extent of different types of perturbations generated by injection. After injecting fluids for some time, pore pressure reaches a certain distance. If the fault is already close to failure with a high prestress level, then it is possible for aseismic slip to propagate beyond pressure diffusion^6,12,13^. However, the extent of shear stress perturbation goes beyond the aseismic slip front, and it is possible for seismicity to be triggered within this region as well^14^. Figure 1b shows how pore pressure and aseismic slip propagate away from the injector over time. At the same time, there exist varying degrees of stress transfer ahead of the aseismic slip front—the closer the distance to the slip front, the higher the stress transfer is, but even a small amount of stress perturbations way ahead of the slip front may have the potential to trigger seismicity.

**Figure 2:**
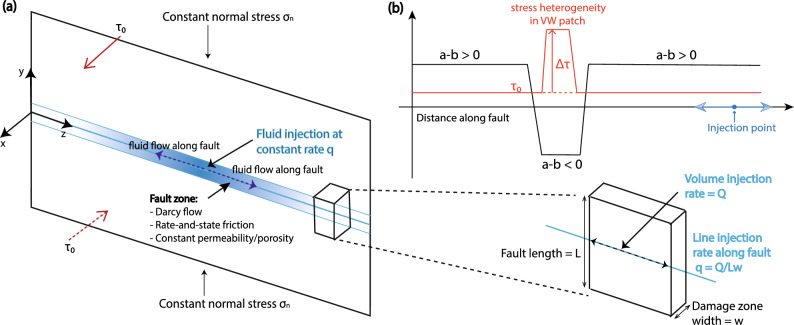
(**a**) The 2D anti-plane problem with fluid injection in the middle of the fault and along-fault Darcy flow through a permeable fault zone with constant porosity and permeability^13^. A volume of fault is drawn to illustrate how to convert the linear injection rate *q* from volumetric injection rate *Q* distributed over surface area $A = Lw$, using $q = Q/A$, by assuming that pressure perturbations are confined to a fault damage zone of width *w* over a length *L*. (**b**) Schematic showing fluid injection into VS part of the fault. As fluid flows along the fault, it encounters a VW patch, in the middle of which exists a stress heterogeneity with elevated prestress $\tau _0 + \Delta \tau$ compared to $\tau _0$ in the rest of the domain.

In this article, we conduct numerical experiments of fluid injection coupled to fault slip in 2D antiplane shear^13^. The fault is governed by rate- and state- friction with the aging law. We inject fluid into a velocity strengthening (VS) part of the fault, and place a velocity weakening (VW) patch some distance away from the injector. Within the VW patch, we further include a stress heterogeneity such that this region starts with a higher shear stress level than the rest of the domain, and therefore is more prone to nucleation (Fig. 2b). We investigate the behavior of this stress heterogeneity over different prestress conditions and fluid injection rates to delineate dominating causes of seismicity in this region. In addition, we quantify the migration speeds of stress transfer that are close to the static triggering thresholds of 0.01 MPa and 0.1 MPa, and compare with that of the aseismic slip front. Finally, we apply insights from this modeling study to seismicity migration patterns in the Weiyuan shale gas field in China.

## Results

### Numerical simulations

The model setup is shown in Fig. 2. We consider a fault with constant normal stress $\sigma _n = 50$ MPa, and constant prestress $\tau _0 = 29.9$ MPa, except at the stress heterogeneity, where we increase the prestress to a higher value $\tau _a = \tau _0 + \Delta \tau$, so that nucleation is more favorable. We selected a high $\tau _0$ so that upon high-rate injection, the VS part of the fault could produce aseismic slip almost immediately. Its initial slip velocity before injection is $\sim 5 \times 10^{-12}$ m/s.

Fluid is injected at a constant rate *q* over a period of 30 days, after which injection is shut off. The way to convert a line injection rate in our model to a volume injection rate is by assuming a fault length of 1 km and width of 1 m (Fig. 2a), so that we can multiply our injection rate by the fault area. For a reference $q = 10^{-5}$ m/s, it will convert to $\sim 26{,}000$ m$^3$/mo, or $\sim 160{,}000$ bbl/mo, close to what is expected of high-rate injection wells^15^. We ignore tectonic loading as we only consider a short period of time. The VW patch is placed from 6 to 8 km away from the injector, and transitions to VS occurs over 500 m on each side of the patch. The stress heterogeneity is placed at the center of the VW patch, with a length of 1 km, exceeding the critical nucleation length given by $L_b = \mu d_c/b\sigma ^{\prime }$^16^, where $\mu$ is the shear modulus, $d_c$ is the state evolution distance, and $\sigma ' = \sigma _n - p$ is the effective normal stress. $L_b \approx 220$ m using the parameters we selected (Table 1). We use a constant porosity $\phi = 0.1$, permeability $k = 10^{-13}$ m$^2$, pore and fluid compressibility $\beta = \beta _\phi + \beta _f = 10^{-8}$ Pa$^{-1}$, and fluid viscosity $\eta = 10^{-3}$ Pa s. This results in a hydraulic diffusivity of $c = k/\phi \beta \eta = 0.1$ m$^{2}$/s, which is consistent with values from many modeling studies related to induced seismicity^17–21^.

**Table 1:** Reference parameters.

Symbol	Description	Value
$\mu$	Shear modulus	32.4 GPa
$\sigma _n$	Fault normal stress	50 MPa
$\tau _a$	Initial shear stress on VW stress heterogeneity	32.7 MPa*
$\tau _0$	Initial shear stress on rest of the fault	29.9 MPa
$f_0$	Reference friction coefficient	0.6
$V_0$	Reference velocity	$10^{-6}$ m/s
*a*	Direct effect parameter	0.01
*b*	State evolution parameter$^{2}$	0.005 (VS), 0.015 (VW)
$d_c$	Characteristic state evolution distance	5 mm
$\Psi _0$	Initial state variable	0.72
$q_0$	Fluid injection rate	$10^{-5}$ m/s*
$\eta$	Fluid viscosity	$10^{-3}$ Pa s
$\beta$	Sum of elastic pore and fluid compressibility	$10^{-8}\,\hbox {Pa}^{-1}$
$\phi$	Porosity	0.1
*k*	Permeability	$10^{-13}$ m$^2$
$L_y$	Domain size in *y* direction	100 km
$L_z$	Domain size in *z* direction	100 km

Values with * are varied in the parameter space study.

**Figure 3:**
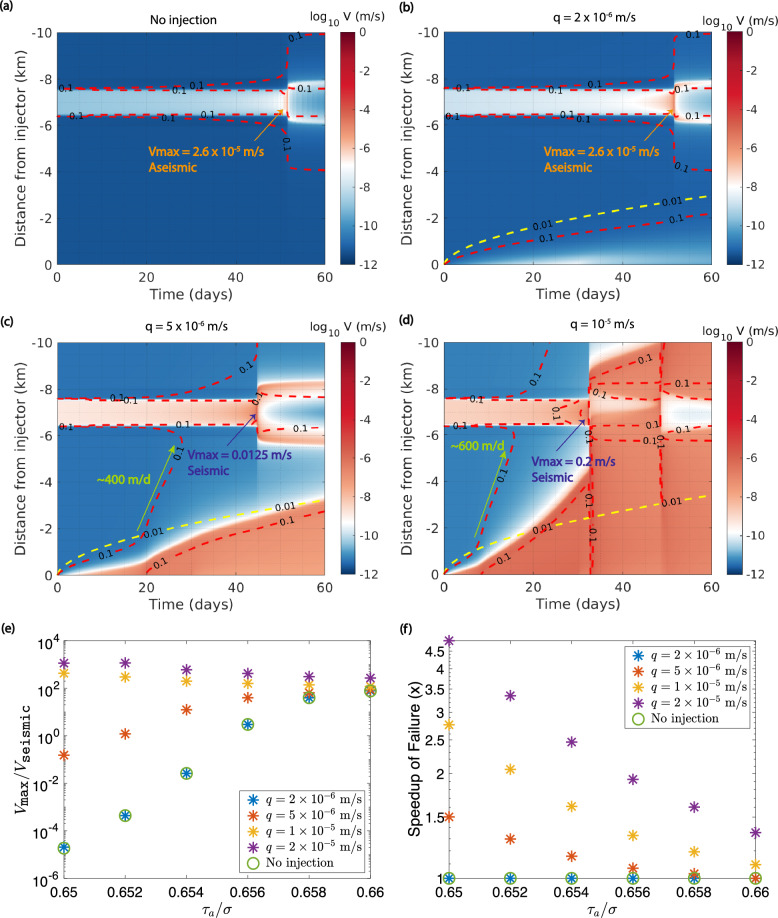
(**a**)–(**d**) Space–time plots of slip velocity for $\tau _a = 32.7$ MPa using different injection rates. The 0.01 MPa pore pressure contour is plotted in yellow dashed line. The 0.1 MPa change in Coulomb failure stress $\Delta CFS$ is plotted in red dashed line. Maximum slip velocity $V_\text {max}$ is indicated on each plot. Migration speed of the 0.1 MPa $\Delta CFS$ is indicated in (**c**) and (**d**). (**e**) $V_{\text {max}}/V_{\text {seismic}}$ and (**f**) speedup in time to failure versus $\tau _a/\sigma$ on this asperity for various injection rates. Note that at low injection rate of $q = 2 \times 10^{-6}$ m/s, the behavior is the same as having no injection.

For a reference case, we consider $\tau _a = 32.7$ MPa, so that $\tau _a/\sigma ' = 0.654$, which is above steady state^22^. Observe the space–time plots of slip velocity over different injection rates in Fig. 3a–d. First, we run a case without any fluid injection (Fig. 3a) to observe how the stress heterogeneity behaves over time. We find that the maximum velocity $V_\text {max}$ at nucleation, which occurs around 51.5 days, is about $2.6 \times 10^{-5}$ m/s. This is below the seismic velocity threshold $V_{\text {seismic}}$ that we set to $10^{-3}$ m/s, and therefore is an aseismic event. We then inject fluid into the fault. At low injection rates, the fault response is not very different from the case without injection. When $q = 2 \times 10^{-6}$ m/s (Fig. 3b), $V_\text {max}$ is exactly the same as for no injection. Slip is negligible and the fault never weakens sufficiently to generate an aseismic slip front. We mark the 0.01 MPa pore pressure diffusion front in yellow dashed line, and the 0.1 MPa change in Coulomb failure stress $\Delta CFS$ in red dashed line, where $\Delta CFS = \Delta \tau + f\Delta (\sigma _n - p) = \Delta \tau + f\Delta p$ in this case, because normal stress $\sigma _n$ is constant. $\Delta \tau$ is the shear stress change and $\Delta p$ is the pore pressure change. Stress transfer is entirely limited within the pore pressure diffusion front, which fails to reach the stress heterogeneity over this period of time.

As we increase the injection rate, however, we begin to observe aseismic slip at higher slip velocities being triggered, causing stress fronts to propagate away from the injector. When $q = 5 \times 10^{-6}$ m/s (Fig. 3c), $V_\text {max} = 0.0125$ m/s, which is already seismic, and nucleation is earlier at about 44.5 days. Note that this occurs after the 30-day injection stops, so it is possible for earthquakes to happen many days after shut-in in our model. Even though the aseismic slip front is still confined within pressure diffusion over this 60-day time period, after $\sim 20$ days, the 0.1 MPa stress transfer front originating from aseismic slip has already accelerated to $\sim 400$ m/day, and reaches the VW stress heterogeneity way before aseismic slip can arrive. Such stress transfer assists the highly stressed region to nucleate both at a higher slip velocity and earlier in time. A further increase in injection rate to $q = 10^{-5}$ m/s (Fig. 3d) increases $V_\text {max}$ to 0.2 m/s, and moves nucleation to 32 days, when the aseismic slip front is still about 2 km away from the nucleation site. At the same time, the migration speed of the 0.1 MPa $\Delta CFS$ increases to $\sim 600$ m/day, and is able to propagate outside of the pressure diffusion front much earlier than for lower injection rates, due to faster propagation of aseismic slip that enables more energetic stress transfer. Figure S1 demonstrates how the stress transfer front precedes the slip front by plotting the time series of $\Delta CFS$ and total accumulated slip at 4 km from the injeciton point. Observe that even though slip is only initiated from 30 days onwards, $\Delta CFS$ starts to become positive as soon as injection begins, and its magnitude reaches the order of 0.1 MPa a little after 10 days. Thus, a positive Coulomb stress transfer initiates way ahead of the onset of significant slip.

The ratio of the maximum slip velocity that the highly stressed VW patch could attain over $V_{\text {seismic}} = 10^{-3}$ m/s is shown in Fig. 3e across different initial stress conditions and injection rates, and Fig. 3f shows the speedup of nucleation (not necessarily seismic) in time compared to having no injection. Notice that at a low injection rate of $q = 2 \times 10^{-6}$ m/s, the behavior is exactly the same as for the case with no injection, indicating the limited extent of stress transfer at low injection rates due to the absence of substantial aseismic slip that could be triggered. In general, higher prestress on the asperity and higher injection rate will result in a higher slip velocity when nucleation occurs. Increasing the injection rate is especially effective when the prestress level is lower, enabling aseismic ruptures to become seismic and greatly reducing the time to reach $V_\text {max}$.

We also note the drastic change in the behavior of this asperity given a very small prestress difference. It is conceivable that across the fault, many such heterogeneous stress asperities exist, and are at different stages of the interseismic phase, some being very close to nucleation. The stress transfer front of 0.1 MPa could therefore be critical to the rupture of these asperities way before the aseismic slip front reaches their vicinity. Many studies have found lower levels of static triggering threshold on the order of 0.01 MPa^23–25^. We propose that the fast propagation of these stress transfer fronts may be the explanation of locally anomalously high seismicity migration rates related to injection.

**Figure 4:**
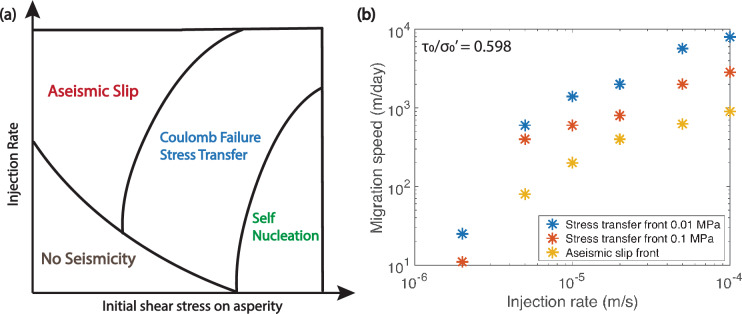
(**a**) Phase diagram of the triggering mechanism of distant seismicity for different initial shear stress on asperity and injection rates. (**b**) Migration speed (m/day) of the 0.01 MPa, 0.1 MPa Coulomb failure stress transfer fronts and the aseismic slip front for different injection rates over an injection period of 30 days.

We summarize a phase diagram delineating the reasons of distant seismicity (i.e. outside of the influence of pore pressure diffusion) being triggered by fluid injection in Fig. 4a. On the lower left hand corner is a region where no seismicity occurs due to low prestress levels on the asperity, and low injection rates that are unable to produce aseismic slip and any significant stress transfer. On the upper left hand corner is seismicity being triggered directly by the arrival of the aseismic slip front. Part of this region still has very low prestress, thus requiring a large stress increase from the slip front in order to nucleate seismically. High injection rates enable the VS part of the fault to weaken sufficiently so as to generate aseismic slip propagation. On the lower right hand corner exist highly-stressed asperities that can nucleate seismically on their own without any stress perturbations from fluid injection, although these ruptures can occur earlier if injection rate is high enough such that significant Coulomb failure stress transfer arrives before the natural nucleation time. In the middle is seismicity triggered by Coulomb failure stress transfer for asperities that either cannot seismically nucleate on their own, but given sufficient stress transfer, rupture as earthquakes before the aseismic slip front arrives; or those that can self-nucleate, but stress transfer advances their time to failure. Therefore, through our numerical experiments, we find many nuances in the mechanisms of injection-induced seismicity. In particular, if the fault has experienced stress perturbations due to injection operations in the past, then it is likely that there exist many highly-stressed asperities which are already close to nucleation, and a small amount of stress transfer from injection-induced aseismic slip could potentially trigger seismicity at a large distance from the injection site. Experimentally, it has also been demonstrated that the limit of the shear stress perturbation is far from the injector, which could promote earthquake nucleation in the neighbouring asperities or segments^14^.

The migration speeds of the 0.01 MPa, 0.1 MPa Coulomb failure stress transfer fronts and the aseismic slip front are shown in Fig. 4b across various injection rates for $\tau _0/\sigma _0' = 0.598$ of the VS part of the fault. If this ratio were lower, the fault will be further from failure, and migration speeds will decrease accordingly^10,13^. We do not consider varying permeability here, as it has been shown that the migration speed will become independent of permeability when the slip front advances well beyond pressure perturbation^26,27^. Notice that the 0.01 MPa stress transfer front is faster than the aseismic slip front by almost an order of magnitude across all injection rates. At the lowest injection rate investigated ($q = 2\times 10^{-6}$ m/s), no aseismic slip front is produced over the injection period, thus its migration speed is zero, and the stress transfer there is purely due to pore pressure diffusion. We note that if the injection period were longer, aseismic slip will be eventually triggered for low injection rates too, but we only consider the fault response with 30 days of injection. On the other hand, at the highest injection rate ($q = 10^{-4}$ m/s), the 0.01 MPa front can reach a speed as high as close to 10 km/day.

### Application to seismicity migration in Weiyuan shale gas field

We now apply what we have learned from the numerical simulations to seismicity migration patterns in the Weiyuan shale gas field. We consider the hydraulic fracturing operations in Shuangshi Town, Zigong City from May 10, 2020 to June 19, 2020 (total 40 days), at the Well Pad Z201H2. The distribution of earthquakes over this entire period is shown in Fig. S2a, which shows that the seismicity is not limited to locations immediately surrounding the horizontal wells, but extends to the east. Reports of fluid leakage in this region suggest that the injected fluid could have diffused to nearby faults that act as high-permeability conduits, therefore resulting in this unusual pattern of seismicity migration. Due to such leakage, the actual fluid rate entering into the modeled fault would be substantially lower than the average total injection rate, which is on the order of $2 \times 10^{-5}$ m/s for this well operation. Figure S2b shows that most of the seismicity is concentrated within 3–4.5 km depth, as higher-permeability limestone and dolomite layers surround the shale layer (Longmaxi formation) in which injections are conducted, but two more low-permeability shale layers above and below act as barriers to fluid flow (see Fig. S3 in Supplementary Information for details of the vertical lithology).

**Figure 5:**
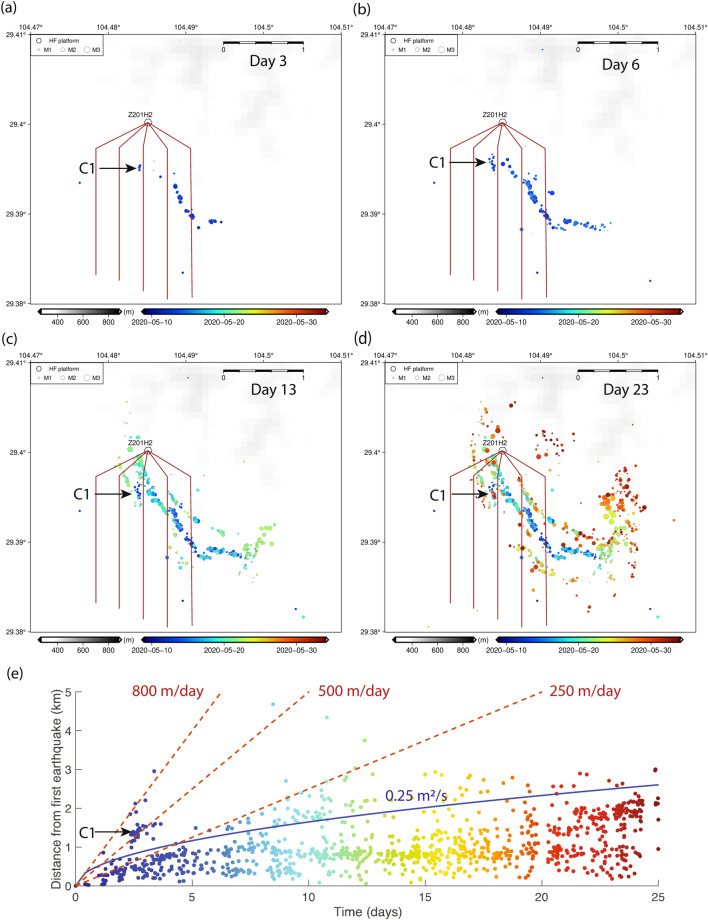
Seismicity induced by hydraulic fracturing operations in Shuangshi Town, Zigong City at the Weiyuan shale gas field in Sichuan Basin, China. (**a**)–(**d**) Map view of seismicity on Day 3, 6, 13 and 23 during hydraulic fracturing, when Well Pad Z201H2 (shown by the hexagon, and horizontal wells are denoted with brown lines extending from the well pad) was in operation. A length bar of 1 km is shown for scale. F1, F2 and F3 denote the three fault segments along which most of the earthquakes occurred during this time. C1 denotes the cluster of seismicity that does not apparently fit the diffusion front, and also migrates faster than most other earthquakes in this sequence. (**e**) Seismicity migration pattern shown as the distance from the first earthquake over time. The blue curve is the pore pressure diffusion front with hydraulic diffusivity 0.25 m$^2$/s. A red dotted line with speed 250 m/day fits earthquakes that migrate faster than the diffusion front after 5 days, and is possibly an indicator of the aseismic slip front. Two more red dotted lines with migration speeds of 500 and 800 m/day are shown to fit seismicity that cannot be explained by either pore pressure diffusion or the aseismic slip front.

Here, we will focus on seismicity evolution over the first 25 days to identify possible driving mechanisms for seismicity migration. We show the map view of earthquakes on Days 3, 6, 13 and 23 in Fig. 5a–d. On Day 3, we observe on Fig. 5a that the fault can already be delineated by some earthquakes that are migrating along its trace. However, there is a large spatial gap between the cluster C1, which is located in the northwest corner of the fault, and the other earthquakes. Such discontinuity does not fit into the usual interpretation of pore pressure diffusion causing earthquake migration. Only on Day 6, as we can see in Fig. 5b, is that spatial gap filled in by other earthquakes that have migrated there possibly due to diffusion. In Fig. 5e, we have plotted the distances of all earthquakes relative to the first earthquake in the sequence over time. We can see that over the first 6 days, a diffusivity of 0.25 m$^2$/s is able to fit most of the seismicity relatively well. This is on the same order of magnitude as the diffusivity we used for our simulations, and is consistent with some estimates of the this region^28,29^. There is also a group of seismicity in Fig. 5e after Day 5 that does not fit under the diffusion curve, and they can be reasonably well fit by a linear migration curve with a speed of 250 m/day. We interpret this as a possible aseismic slip front migration. However, the earthquakes in C1 are still outliers due to their fast propagation speed of more than 500 m/day. We have produced the magnitude-time plot of events during days 2–4 that are at least 1 km away from the origin, which includes this cluster (Fig. S5). It can be seen that for this cluster, the events do not exhibit typical characteristics of a mainshock–aftershock sequence, in that we don’t observe an event of magnitude much greater than subsequent ones. Therefore, this cluster consists of independent events being triggered by a long-range stress transfer mechanism. There are a few other earthquakes that are triggered at even farther locations, and they can be mostly fit by an 800 m/day migration speed. If we were to fit all of them under a diffusion curve, the diffusivity would have to exceed 2 m$^2$/s, which would overshoot the fitting of the rest of the earthquakes by a large amount, so a larger diffusivity is not a reasonable explanation according to the seismicity evolution we observe from Fig. 5a, b. Therefore, we see these outlying earthquakes as being triggered by stress transfer in the far-field that is potentially caused by aseismic slip, even when the aseismic slip itself has not reached that distance in such a short period of time. We interpret these faster migration fronts as shear stress fronts with varying magnitude of stress perturbation that are sufficient to trigger seismicity in this region, without requiring the aseismic slip front itself to arrive.

Relating this to Fig. 4b, we see that the aseismic slip front migration speed of 250 m/day puts the injection rate between $10^{-5}$ and $2 \times 10^{-5}$ m/s, which is close to the reported injection rate when taking into account some fluid leakage. At the inferred actual fluid flow rate into the fault, the 0.1 MPa stress transfer front would have a migration speed of $\sim 500$ m/day, and the 0.01 MPa front a speed of $\sim 1000$ m/day. Thus, the fast 500 m/day migration of seismicity in C1 could potentially be attributed to the 0.1 MPa stress transfer to highly-stressed asperities in that area.

## Discussion

As we observe from the simulations, a wide range of stress transfer front migration speeds exist, depending on fault stress conditions and injection rates. The higher the prestress on the fault, and the higher the injection rate, the faster stress transfer could propagate. In addition, such stress transfer can cause previously aseismic nucleation to become seismic in nature, as well as advance the timing of the event. Meanwhile, below a certain injection rate, no appreciable aseismic slip will be even triggered during the operation period, precluding shear stress transfer due to aseismic slip, such that pore pressure diffusion is the dominant mechanism for earthquake triggering. This would greatly limit the spatial extent of seismicity. Given the difficulty of knowing the stress condition on the fault before injection operations, it would be advisable to control the injection rate to have a better control over induced seismicity^30–35^. Additionally, in-situ tests such as the Seismogenic Fault Injection Test (SFIT) have been proposed to calibrate the seismic response to fluid injection of known faults, so as to better understand the risk associated with seismicity trailing an anthropogenic operation^36^.

As this study has demonstrated, different triggering mechanisms of seismicity are possible, and one should not use a single approach when evaluating injection related earthquakes. For example, the apparent hydraulic diffusivity inferred from seismicity migration away from the injector could be deceptive if we only consider pore pressure diffusion. If faster migration is caused by stress transfer mechanisms and not fluid diffusion, the apparent hydraulic diffusivity will be vastly overestimated to encompass those events. Moreover, it is also insufficient to attribute any linear migration trend only to the aseismic slip front, since propagating stress fronts with smaller shear stress perturbations could also trigger seismicity in its path and explain unusually high seismicity migration rates of earthquake clusters that do not fit the overall aseismic slip migration trend.

We note that on our modeled fault, which is in antiplane strain, fault slip induces no change in normal stress, so there is no poroelastic effect, which is arguably an important elastic stress transfer mechanism that is responsible for long-range triggering of seismicity. We choose to focus here on the effect of stress transfer originating from injection-induced aseismic slip and observe whether this alone could possibly explain seismicity that is triggered far from the injection site. However, it would be important to incorporate poroelasticity in a future study of a fault in plane strain, so we can study the stress transfer contributions from both poroelastic effects and aseismic slip.

Finally, this study may also be applicable to explaining distant or delayed earthquake triggering related to injection operations. It is possible for earthquakes to occur in regions tens of kilometers away from the injection site due to the fast propagation of stress transfer fronts. In addition, Coulomb stress transfer need not be confined to a single fault, but could potentially also trigger off-fault seismicity^3,37^. If the stress conditions are right, a small amount of perturbation like 0.01 or 0.1 MPa could cause a seismic nucleation. In addition, the stress fronts can keep propagating even after injection stops, as aseismic slip does not cease at that point in time, and its arrest is highly depend on the initial fault stress criticality and the pressurization duration^38^. Pore pressure diffusion also continues to expand until equilibrium. Thus it is still possible for seismicity to be triggered after the shut-in of wells due to a combination of the above factors that causes a positive change in the Coulomb failure stress^39–41^.

## Methods

### Coupled fault and fluid model

The simulations use a 2D coupled fault slip and pore pressure diffusion numerical model^13,21^. The fault is located at $y=0$, and displacements *u*(*y*, *z*, *t*) are in the *x*-direction. We assume symmetry about the fault, enabling us to model only half the domain ($y\ge 0$). The governing equations for quasi-static antiplane shear deformation of an elastic solid are:

1 $$\begin{aligned} \frac{\partial \sigma _{xy}}{\partial y} + \frac{\partial \sigma _{xz}}{\partial z} = 0, \quad \sigma _{xy} = \mu \frac{\partial u}{\partial y}, \quad \sigma _{xz} = \mu \frac{\partial u}{\partial z}, \end{aligned}$$

where $\sigma _{xy}$ and $\sigma _{xz}$ are the quasi-static stress changes associated with displacement *u* and $\mu$ is the shear modulus, which we assume is constant. We define slip and slip velocity as:

2 $$\begin{aligned} \delta (z,t) = 2u(0,z,t) \quad \text{ and } \quad V(z,t) = \partial \delta / \partial t, \end{aligned}$$

respectively. The fault boundary conditions are

3 $$\begin{aligned} \tau = f(\Psi ,V) \left( \sigma _0^{\prime } - p\right) , \end{aligned}$$

where $\tau (z,t)$ is the shear stress and $\Psi (z,t)$ is the state variable. Equation (3) sets the shear stress equal to the frictional strength, where $f(\Psi ,V)$ is the rate- and state- friction coefficient, $\sigma _0^{\prime }$ is the initial effective normal stress, and *p*(*z*, *t*) is the change in pore pressure.

We use the quasi-dynamic approximation with radiation damping^42^:

4 $$\begin{aligned} \tau (z,t) = \tau _0 + \sigma _{xy}(0,z,t) - \eta _{rad} V, \end{aligned}$$

where $\tau _0$ is the initial shear stress and $\eta _{rad} = \rho c / 2$ is the radiation damping parameter, with $c = \sqrt{\mu / \rho }$ being the S-wave speed.

For the computation of the rate- and state- friction coefficient, we use the regularized form^43^:

5 $$\begin{aligned} f(\Psi ,V) = a\sinh ^{-1} \left( \frac{V}{2V_0}e^{\Psi /a} \right) , \end{aligned}$$

where *a* is the direct effect parameter, $V_0$ is the reference velocity, and $f_0$ is the reference friction coefficient. We use the aging law for state evolution^44,45^:

6 $$\begin{aligned} \frac{\hbox {d}\Psi }{\hbox {d}t} = \frac{bV_0}{d_c}(e^{(f_0-\Psi )/b} - \frac{V}{V_0}). \end{aligned}$$

The computational domain has three other boundary conditions:

7 $$\begin{aligned} \sigma _{xz}(y,0,t) = 0, \quad \sigma _{xz}(y,L_z,t) = 0, \quad u(L_y,z,t) = 0, \end{aligned}$$

where $L_y$ and $L_z$ are the domain dimensions. The first two conditions indicate traction-free boundaries perpendicular to the fault, and the zero-displacement condition on the remote boundary parallel to the fault indicates that there is no tectonic loading. Since we are considering a short time interval, effects from plate loading can be ignored. We use a sufficiently large domain to ensure that the solution is relatively insensitive to conditions applied on remote boundaries.

Our idealized fluid transport model accounts only for along-fault flow and pressure diffusion^46–50^. This is motivated by the commonly observed fault zone structure of a permeable damage zone embedded within relatively impermeable host rock^51,52^. Darcy velocity *q* is given by:

8 $$\begin{aligned} q = -\frac{k}{\eta }\frac{\partial p}{\partial z}, \end{aligned}$$

where *k* is the permeability, $\eta$ is the fluid viscosity.

The fluid mass conservation equation is:

9 $$\begin{aligned} \phi \beta \frac{\partial p}{\partial t} = \frac{\partial }{\partial z} \left( \frac{k}{\eta }\frac{\partial p}{\partial z} \right) + q_0 \delta _D(z), \end{aligned}$$

where $q_0$ is a constant injection rate, and $\delta _D(z)$ is the Dirac delta function that places the source at $z=0$. This is a diffusion equation with hydraulic diffusivity $c = k / (\phi \beta \eta )$.

Finally, we use a high-order SBP-SAT finite difference method for spatial discretization along with adaptive time stepping, with error control on slip and the state variable^53–55^. Pressure is solved implicitly using backward Euler, while slip and state variable are solved explicitly with an adaptive Runge–Kutta method^49^. The solution of pressure at every time step updates the effective normal stress on the fault.

### Earthquake catalog

The initial earthquake catalog, which includes more than 32,000 events in the magnitude range $M_L$ 0.0–$M_w$ 5.0 with a magnitude completeness of $M_L$ 1.5 and spans from 1st March 2019 to 28th February 2021, is produced using the Sichuan Earthquake Agency network, which possesses 21 stations within 30 km of the Weiyuan Shale Gas Block. The detection method is STA/LTA, and P and S phases are picked manually. A grid search was conducted to provide initial locations, and the absolute locations were further refined by decreasing travel time residuals using a local 1-D velocity model.

We first refined the absolute earthquake locations using the absolute travel times recorded by a well-covered seismic network (Fig. S4b). We then conducted double-difference earthquake relocation using 3-D velocity models and waveform cross-correlation constraints, which leads to tremendously improved earthquake locations. The waveform cross-correlation method is applied to improve differential travel times so as to obtain high-resolution relative earthquake locations. Window lengths of 1.5 s (0.5 s before and 1.0 s after) and 4 s (1 s before and 3 s after) are applied to P and S waves respectively for three-component waveform cross-correlation. Double-difference earthquake relocation^56^ with cross-correlation differential times was conducted using the tomoDD program^57^ with an improved local 3D velocity structure^58^. We conducted bootstrap analysis by randomly removing differential times during double-difference for 1000 times, which leads to statistical location errors of 50 m in the horizontal direction and 80 m in depth. Therefore, it is highly unlikely that the observations in the Shuangshi cluster are artifacts from earthquake relocation. This judgment is further supported by the same time-spatial pattern presented in absolute and relative locations (Fig. S4b,c) and linearized seismicity in other locations (Fig. S4a).

The magnitudes of earthquakes recorded by our catalog are all above $M_L$ 0.0 (Fig. S4d), which is above the general magnitude range of $M_L$ $-2.0$ to $M_L$ 0.0 for microseismicity directly generated by hydraulic fracturing. Therefore, these events correspond to the reactivation of seismicity on faults in the research area. The Weiyuan area experienced multi-stage tectonic evolution in geological history, including the NE-striking basement rift developed in the Neoproterozoic (red dashed line in Fig. S4a) that was inferred to influence sedimentary layers due to uneven solidification^58^, and subsequent far-field effects of tectonic events in the northwestern and western peripheries of the Sichuan Basin. These led to the structural deformation of the Weiyuan anticline (NE-striking) in 38 Ma^59^ and the current east–west SHmax orientation^60^. Therefore, it is possible that there exist faults with different orientations in the Weiyuan area. In addition, geological surveys reveal the existence of NNW-SSE faults that extend to the surface in the area (Fig. S4a), which further indicates local complex structures. We infer that the spatiotemporal pattern of seismicity in the Shuangshi area is due to the reactivation of faults with different orientations.

### Model parameters

The parameters used in this study are shown in Table 1. Most parameter values are chosen to be consistent with those in.

### Supplementary Information

Supplementary Information.

## Data Availability

The simulation data in this study are available in Open Science Framework: https://doi.org/doi:10.17605/OSF.IO/68X43.

## Code availability

All simulations were performed in the open-source code Scycle-2, available at https://bitbucket.org/yyy910805/scycle-2.
