# Author Correction: Stress transfer outpaces injection-induced aseismic slip and triggers seismicity

**DOI:** 10.1038/s41598-023-45367-x

**Published:** 2023-11-10

**Authors:** Yuyun Yang, Hongfeng Yang, Jinping Zi

**Affiliations:** 1 Earth and Environmental Sciences Programme, The Chinese University of Hong Kong, Shatin, Hong Kong; 2 Shenzhen Research Institute, The Chinese University of Hong Kong, Shenzhen, China; 3 Institute of Environment, Energy and Sustainability, The Chinese University of Hong Kong, Shatin, Hong Kong

Correction to: *Scientific Reports* 10.1038/s41598-023-43760-0, published online 03 October 2023

The original version of this Article omitted an affiliation, Shenzhen Research Institute, for Hongfeng Yang. The correct affiliations are listed below.

^1^Earth and Environmental Sciences Programme, The Chinese University of Hong Kong, Shatin, Hong Kong

^2^Shenzhen Research Institute, The Chinese University of Hong Kong, Shenzhen, China

^3^Institute of Environment, Energy and Sustainability, The Chinese University of Hong Kong, Shatin, Hong Kong

In addition, the Acknowledgements section was incorrect.

“We appreciate constructive comments and suggestions from two anonymous reviewers. We thank E.M. Dunham from Stanford University for insightful discussions. Y.Y. is supported by the Research Grants Council Postdoctoral Fellowship, University Grants Committee, Hong Kong (PDFS2223-4S08). H.Y. is supported by the Research Grants Council, University Grants Committee, Hong Kong (No. 14303721) and the National Natural Science Foundation of China (U2139203). All authors are supported by the Faculty of Science at the Chinese University of Hong Kong.”

now reads:

“We appreciate constructive comments and suggestions from two anonymous reviewers. We thank E.M. Dunham from Stanford University for insightful discussions. H.Y. is supported by the National Natural Science Foundation of China (No. U2139203) and the Research Grants Council, University Grants Committee, Hong Kong (No. 14303721). Y.Y. is supported by the Research Grants Council Postdoctoral Fellowship, University Grants Committee, Hong Kong (PDFS2223-4S08). J.Z. is supported by the Hong Kong PhD Fellowship Scheme. All authors are supported by the Faculty of Science at the Chinese University of Hong Kong.”

The original Article has been corrected.

