# Supplementary material for: Stress transfer outpaces injection-induced aseismic slip and triggers seismicity
Source: Sci Rep. 2023 Oct 3;13:16626. doi: 10.1038/s41598-023-43760-0 (PMC10547725; doi:10.1038/s41598-023-43760-0)
Supplement: Supplementary file 1 — Supplementary Information. [file 41598_2023_43760_MOESM1_ESM.pdf]

Supplementary Information for

Stress Transfer Outpaces Injection-Induced Aseismic Slip and Triggers Seismicity

Yuyun Yang[1], Hongfeng Yang[1]*, Jinping Zi[1]

[1]Earth and Environmental Sciences Programme, The Chinese University of Hong Kong, Hong Kong SAR

*Corresponding author. Email: hyang@cuhk.edu.hk

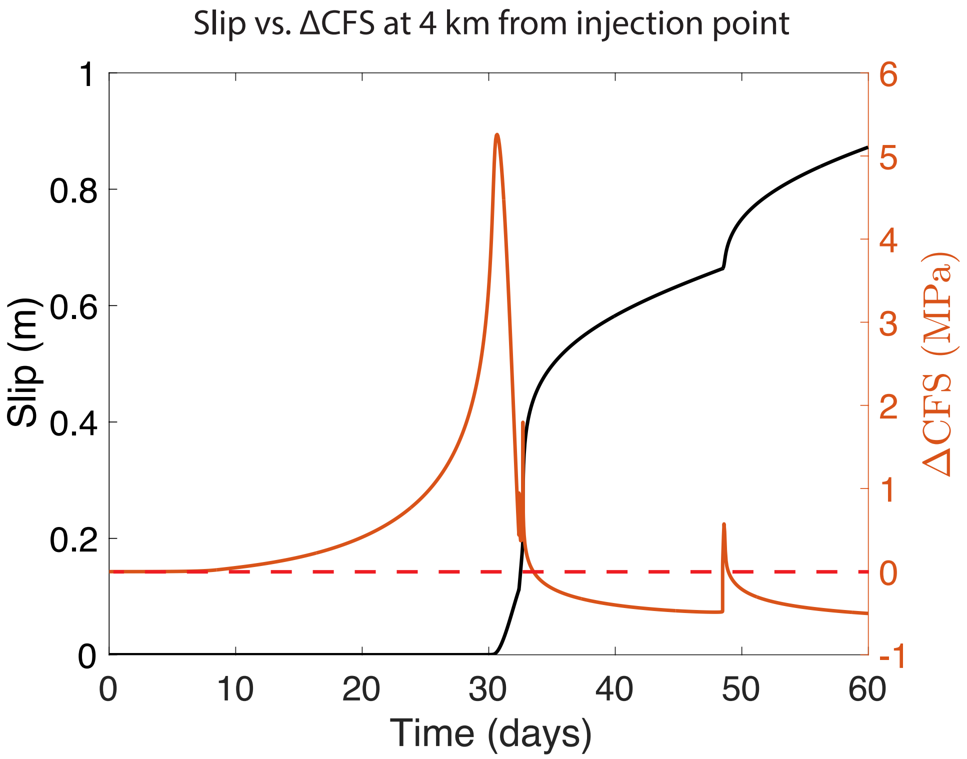

Fig. S1. Total accumulated slip (black) and ΔCFS (red) at 4 km from the injection point for the case in Fig. 3d ($q = 10^{-5}$ m/s). Observe that even though slip is only initiated from ~30 days onwards, ΔCFS starts to become positive as soon as injection begins, and its magnitude reaches the order of 0.1 MPa a little after 10 days. Thus, a positive Coulomb stress transfer initiates way ahead of the onset of significant slip.

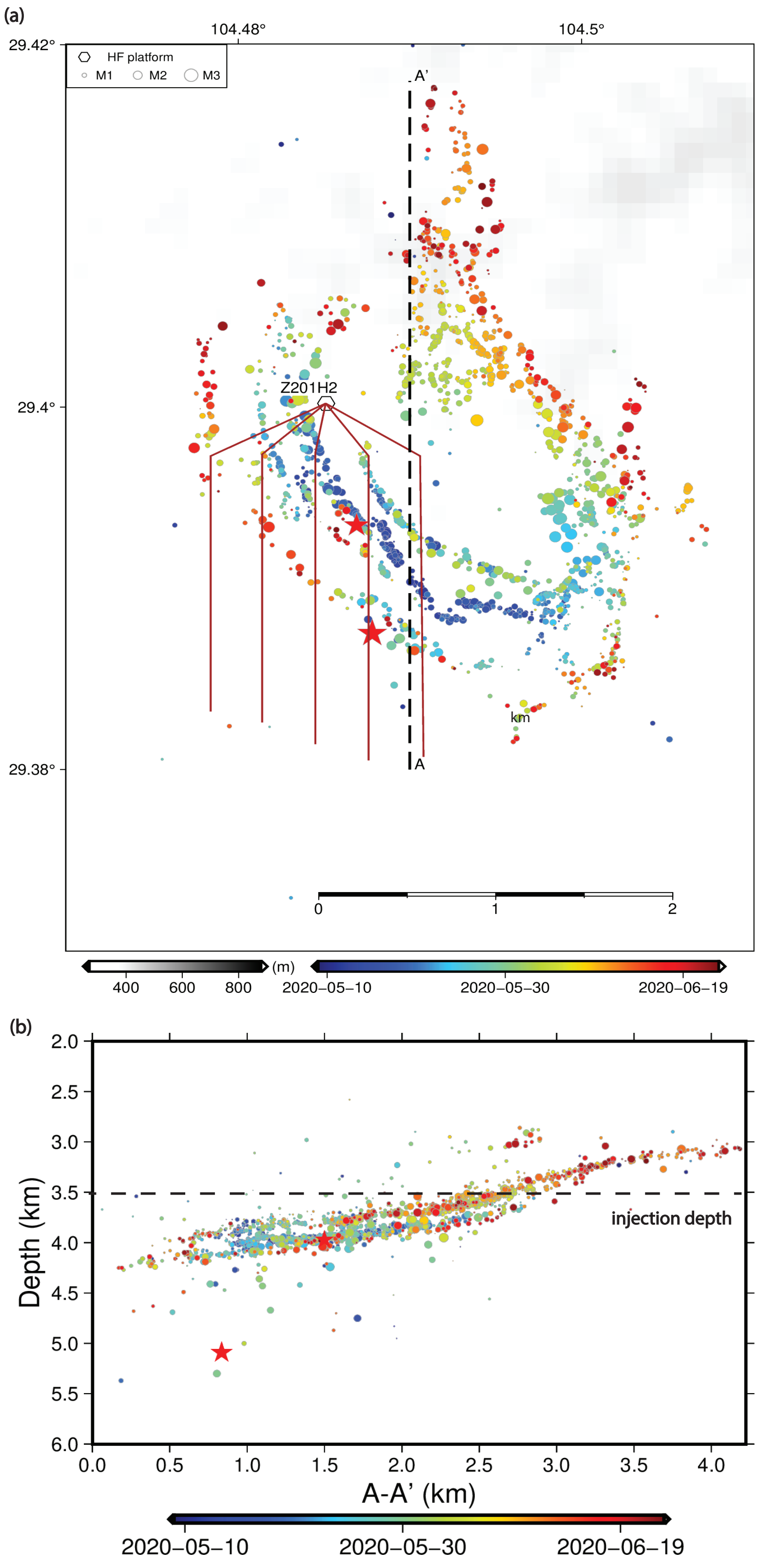

Fig. S2. (a) Map view of earthquakes over May 10, 2020 – Jun 19, 2020 induced by hydraulic fracturing operations in Shuangshi Town, Zigong City at the Weiyuan Shale Gas field in Sichuan

Basin, China when Well Pad Z201H2 (shown by the hexagon, and horizontal wells are denoted with brown lines extending from the well pad) was in operation. A length bar of 2 km is shown for scale. Two Mw > 3.0 events are shown in red stars. (b) Depth distribution of earthquakes along the A-A' transect in (a). The injection depth of ~3.5 km is shown. An Mw > 3.0 event over 5 km deep occurs near the Weiyuan Anticline and could be an indication of a basement fault in that region being reactivated by injection. The other Mw > 3.0 event occurred 3 days after shut-in.

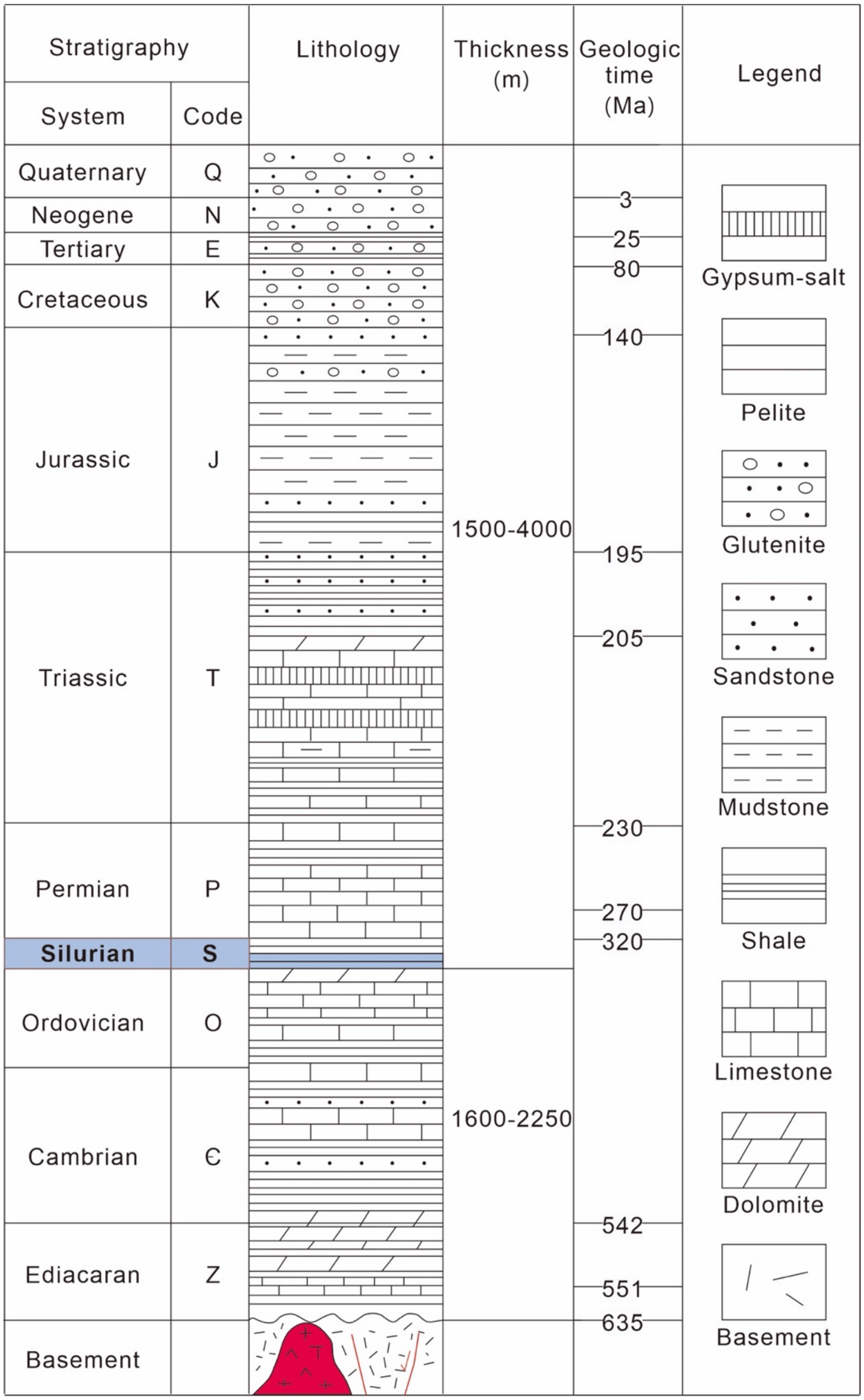

Fig. S3. Vertical lithology of Weiyuan Shale Gas Field. Hydraulic fracturing is done in the Longmaxi formation (highlighted in purple) in the Silurian basin. Limestone and dolomite layers above and below the Longmaxi formation could act as higher permeability conduits for fluid flow, before encountering more shale layers in the Permian and Ordovician basins that act as permeability barriers. This explains the concentration of earthquakes at certain depths in Fig. 5b.

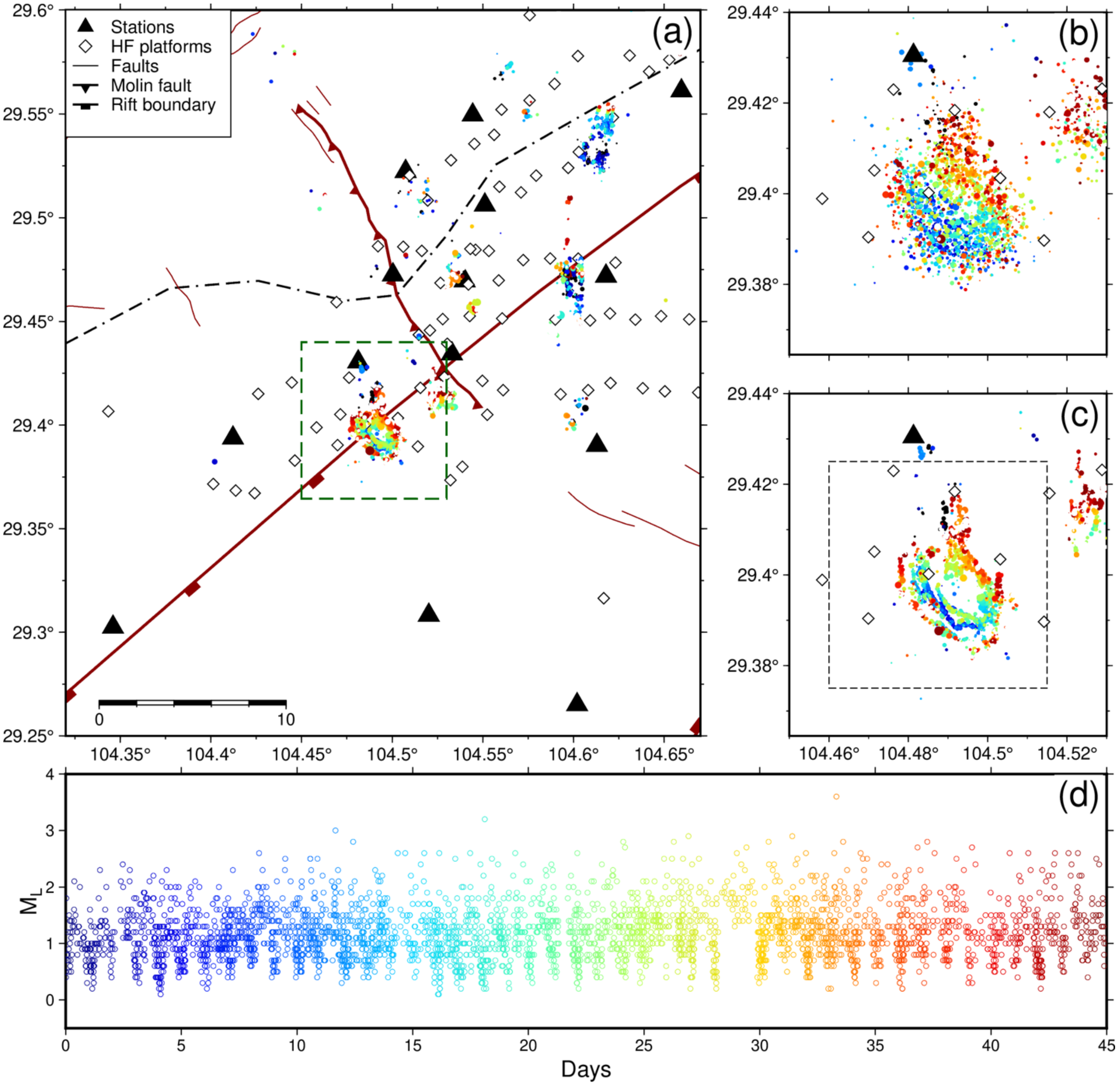

Fig. S4. Earthquake catalog in the Weiyuan area for the period May to June 2020. (a) Map view plot showing the distribution of relocated earthquakes and the seismic network (black triangles). The dashed rectangle denotes the plot area of (b) and (c) with the Shuangshi cluster in the center. The squares indicate HF platforms. (b) Absolute earthquake location results using HYPOINVERSE (Klein, 2002) with 1D velocity models. (c) Double-difference relocation results using 3D velocity models and waveform cross-correlation constraints. (d) Magnitude-Time plot of the Shuangshi cluster from the dashed box region in (c).

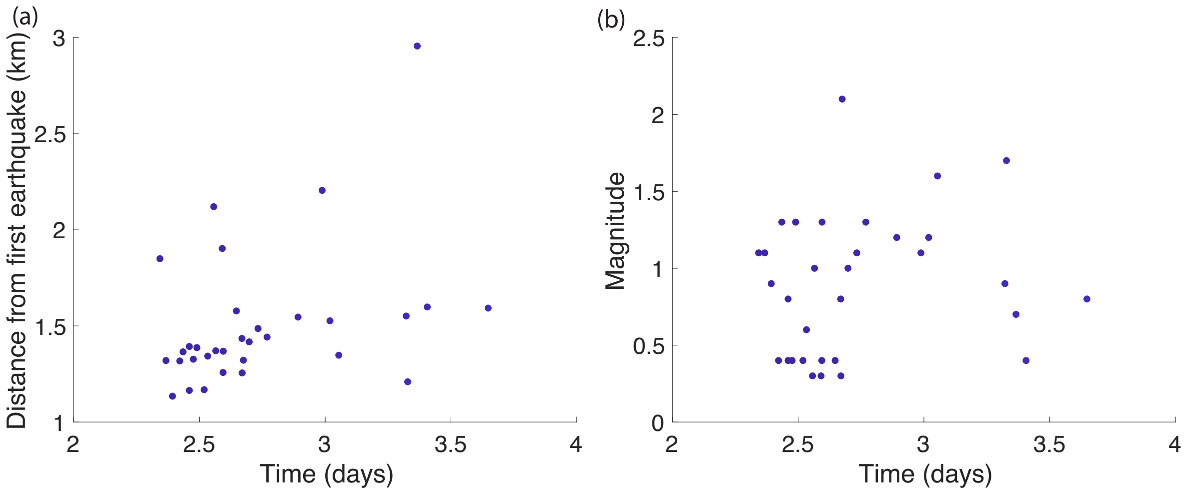

Fig. S5. A zoom-in view of events from Days 2-4 that are at least 1 km away from the first earthquake in the sequence. (a) Distance vs. time plot. (b) Magnitude vs. time plot. There is no clear mainshock-aftershock sequence, therefore we consider them to be standalone earthquakes.
